# Effect of Chlorhexidine and Tea Tree Oil on Reducing the Number of Oral Microorganisms

**DOI:** 10.1055/s-0043-1769900

**Published:** 2023-08-02

**Authors:** André Luiz de Melo Moreno, Clóvis Lamartine de Moraes Melo Neto, Marcelo Coelho Goiato, Nathaly Vilene de Araujo Moreno, Daniela Micheline dos Santos, Cássia Cunha de Lima, Rogério Heládio Lopes Motta, Juliana Cama Ramacciato

**Affiliations:** 1 Department of Dental Materials and Prosthodontics, São Paulo State University (UNESP), School of Dentistry, Araçatuba, São Paulo, Brazil; 2 Oral Oncology Center, São Paulo State University (UNESP), School of Dentistry, Araçatuba, São Paulo, Brazil; 3 Courses for Dentistry (Instituto de Excelência em Ensino e Pesquisa), Manaus, Amazonas, Brazil; 4 Area of Pharmacology, Anesthesiology and Therapeutics, São Leopoldo Mandic University, School of Dentistry, Campinas, São Paulo, Brazil

**Keywords:** chlorhexidine, tea tree oil, dental plaque, dental implants, mouthwashes, *Streptococcus mutans*, Melaleuca

## Abstract

**Objectives** Thus, the aim of this study was to compare the effect of using two preoperative mouthwashes (0.12% chlorhexidine and 0.2% tea tree oil) on the number of colonies of oral microorganisms.

**Materials and Methods** Forty participants who needed to be rehabilitated with dental implants were included in this study. They were randomly divided into two groups (chlorhexidine group and tea tree group; *n* = 20, each). For each group, saliva samples were collected at four different times: T0 (initially)—before using the mouthwash, T1—after 1 minute of using the mouthwash, T10—after 10 minutes of using the mouthwash, and T60—after 60 minutes of using the mouthwash. At T0 and T1, saliva samples were collected before implant placement surgery, and at T10 and T60, saliva samples were collected during surgery. In each group, one saliva sample was collected at each evaluated time point for each patient, totaling 4 saliva collections per patient. MSB agar (Mitis-Salivarius-Bacitracin) and BHI agar (Brain Heart Infusion) culture media were used in each group. Microbial colony counts were performed using a magnifying glass and recorded in CFU (colony forming units)/mL. Statistical analyses were performed using the Friedman, Mann–Whitney U and Wilcoxon tests ( *p* < 0.05).

**Results** Based on MSB agar culture medium, at T0, the number of *Streptococcus mutans* colonies in the chlorhexidine group was significantly higher compared with the tea tree group ( *p* <0.05; MSB agar). The chlorhexidine group showed significantly lower CFU/mL values for *Streptococcus mutans* at T1, T10, and T60 compared with the tea tree group ( *p* <0.05; MSB agar). Based on BHI agar culture medium, at T0, the chlorhexidine group showed a significantly lower value of CFU/mL compared with the tea tree group ( *p* < 0.05; BHI agar). At T1, T10, and T60, the chlorhexidine group showed significantly lower CFU/mL values compared with the tea tree group ( *p* <0.05; BHI agar).

**Conclusion** Chlorhexidine is more indicated as a preoperative mouthwash than tea tree oil, due to its significantly more effective antimicrobial action.

## Introduction

Biofilm is the main etiological factor for the development of peri-implantitis, which can lead to failure of osseointegration of dental implants. 1 2 A mouthwash is commonly used before dental implant placement to reduce the number of oral microorganisms that cause peri-implantitis. The purpose of this is to help prevent an infection in the tissues around the implant after it is placed.

Tea tree oil (or *Melaleuca alternifolia* oil) is extracted from the leaves and terminal branches of a native Australian plant called *Melaleuca alternifolia* by steam and vacuum distillation processes. 3 This oil has antimicrobial and anti-inflammatory actions due to terpinen-4-ol and α-terpineol, which are part of its composition. 4 Thus, the applicability of tea tree oil has been studied in dentistry, for the treatment of gingivitis and periodontitis and control of plaque index, 5 6 7 8 and in medicine. 3 9

Chlorhexidine (cationic bisbiguanide) is considered a gold standard antimicrobial agent. 2 10 11 12 However, its prolonged use can cause dental dyschromia, taste alteration, and increased formation of dental calculus. 2 7 10 11 13 Based on this, studies have been carried out to verify whether tea tree oil is an efficient substitute for chlorhexidine, and the results of using this oil have been promising. 7 8 10 11

In 2023, a search in the PubMed database using the terms “chlorhexidine” and “tea tree oil” or “melaleuca alternifolia oil” showed that there are no articles comparing these two solutions, based on colony counts of oral microorganisms, before and during surgery for implant placement. Thus, the aim of this study was to compare the effect of using two preoperative mouthwashes (0.12% chlorhexidine and 0.2% tea tree oil) on the number of colonies of oral microorganisms.

## Materials and Methods

### Ethics Committee

This study was approved by the Human Research Ethics Committee of the São Leopoldo Mandic University (n ^o^ 2009/0248) and was carried out in accordance with the Declaration of Helsinki. 14 Forty participants who met the inclusion and exclusion criteria were included in this study.

### Inclusion Criteria

➢ Men and women between 20 and 50 years old who need to be rehabilitated with dental implants.
➢ Partially dentate maxilla and/or mandible.
➢ American Society of Anesthesiologists (ASA) I and ASA II (controlled systemic disease). 15
➢ Absence of oral diseases (e.g., periodontitis, gingivitis, and caries). 16
➢ One month without the use of oral antimicrobial mouthwashes and antibiotics before surgery. 17

### Exclusion Criteria

➢ Complete edentulism.
➢ Those who refused to participate in the study.

### Groups

The 40 patients were randomly divided into two groups ( *n* = 20, each):

➢ Chlorhexidine group—use of 0.12% chlorhexidine gluconate before implant placement surgery 2 8 (Periogard, Colgate, Brazil).
➢ Tea tree group—use of 0.2% tea tree oil 8 before implant placement surgery (Proderma—Manipulation Pharmacy of Piracicaba, Brazil). The composition of the mouthwash in this group was as follows: *Melaleuca alternifolia* oil (20%), Tween 80 (1.32g), 8 and purified water qsp (330 mL).

In each group, the oral rinse time was 2 minutes. The same toothpaste (Colgate, Colgate Máxima Proteção Anticáries, Brazil) was used by all participants, in both groups, for 1 month before surgery. 8 This was done to prevent participants from using toothpastes with different levels of antimicrobial efficacy.

MSB agar (Mitis-Salivarius-Bacitracin) and BHI agar (Brain Heart Infusion) culture media were used in each group.

### Sample Collection

Saliva collections began after patients had fasted for 60 minutes. For each group, saliva samples were collected at four different times:

➢ T0 (initially)—before using mouthwash.
➢ T1—after 1 minute of using the mouthwash.
➢ T10—after 10 minutes of using the mouthwash.
➢ T60—after 60 minutes of using the mouthwash.

At T0 and T1, saliva samples were collected before implant placement surgery, and at T10 and T60, saliva samples were collected during surgery. The total amount of saliva collected from each participant at each time point was 2 mL. At each evaluation time, the patient's saliva was stored in a new sterile Eppendorf tube. 8 Thus, four tubes were collected from each participant in both groups (T0, T1, T10, and T60). Subsequently, these tubes were stored in a freezer for 24 hours before laboratory evaluations.

### Laboratory Preparation of Samples

Samples were diluted 100 times in sterile saline. 8 After dilution, an aliquot of 10 μL was seeded in two Petri dishes (5 × 2 cm), containing 5mL of sterile MSB agar in each one of them, to count Streptococcus mutans colonies. Furthermore, another 10μL aliquot was seeded in two Petri dishes (5 × 2 cm), containing 5mL of sterile BHI agar in each one of them, to count the colonies of all microorganisms that can grow in this medium. Petri dishes were placed in an incubator with 10% CO _2_ at 37°C for 48 hours. 8 Microbial colony counts were performed using a magnifying glass and recorded in CFU (colony forming units)/mL.

### Statistical Analysis

Statistical analysis was performed using the SPSS software (Statistical Package for the Social Sciences—version 24.0, IBM Corp, New York, United States). Friedman, Mann–Whitney U, and Wilcoxon tests were performed ( *p* < 0.05).

## Results

### Chlorhexidine Group (MSB Medium)

**Fig. 1:**
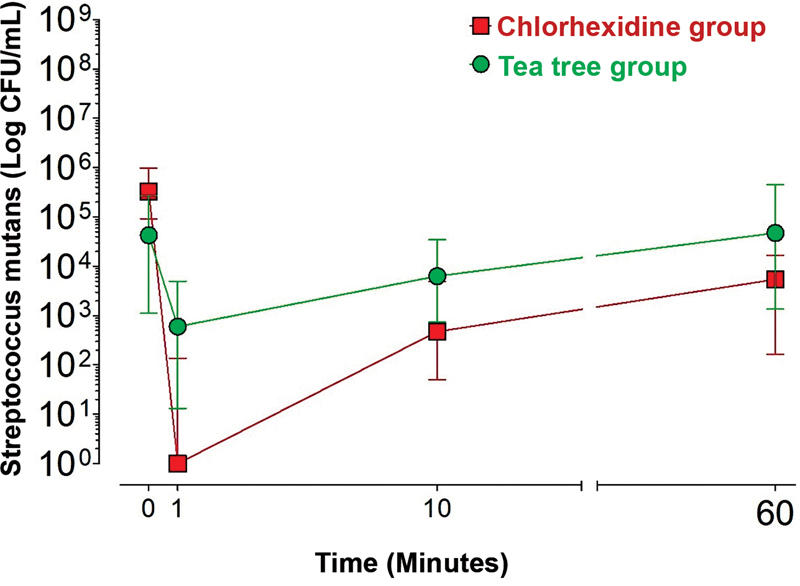
the median values of colony forming unit (CFU/mL) of *Streptococcus mutans* (Mitis-Salivarius-Bacitracin medium) according to treatment and time.

In the chlorhexidine group, there was a significant decrease in the number of *Streptococcus mutans* colonies at T1, T10, or T60 compared with T0 ( *p* < 0.05). There was no significant difference between T1 and T10 ( *p* > 0.05). The number of *Streptococcus mutans* colonies at T1 was significantly lower than at T60 ( *p* < 0.05; Fig. 1 ).

### Tea Tree Group (MSB Medium)

In the tea tree group, there was a significant reduction in the number of *Streptococcus mutans* colonies at T1 when compared with T0 ( *p* < 0.05). There were no significant differences between T0, T10, and T60 ( *p* > 0.05). At T1, there was a significant reduction in the number of *Streptococcus mutans* colonies compared with T10 or T60 ( *p* <0.05; Fig. 1 ).

### Chlorhexidine Group versus Tea Tree Group (MSB Medium)

At T0, the number of *Streptococcus mutans* colonies in the chlorhexidine group was significantly higher compared with the tea tree group ( *p* < 0.05). The chlorhexidine group showed significantly lower CFU/mL values for *Streptococcus mutans* at T1, T10, and T60 compared with the tea tree group ( *p* < 0.05; Fig. 1 ).

### Chlorhexidine Group (BHI Medium)

**Fig. 2:**
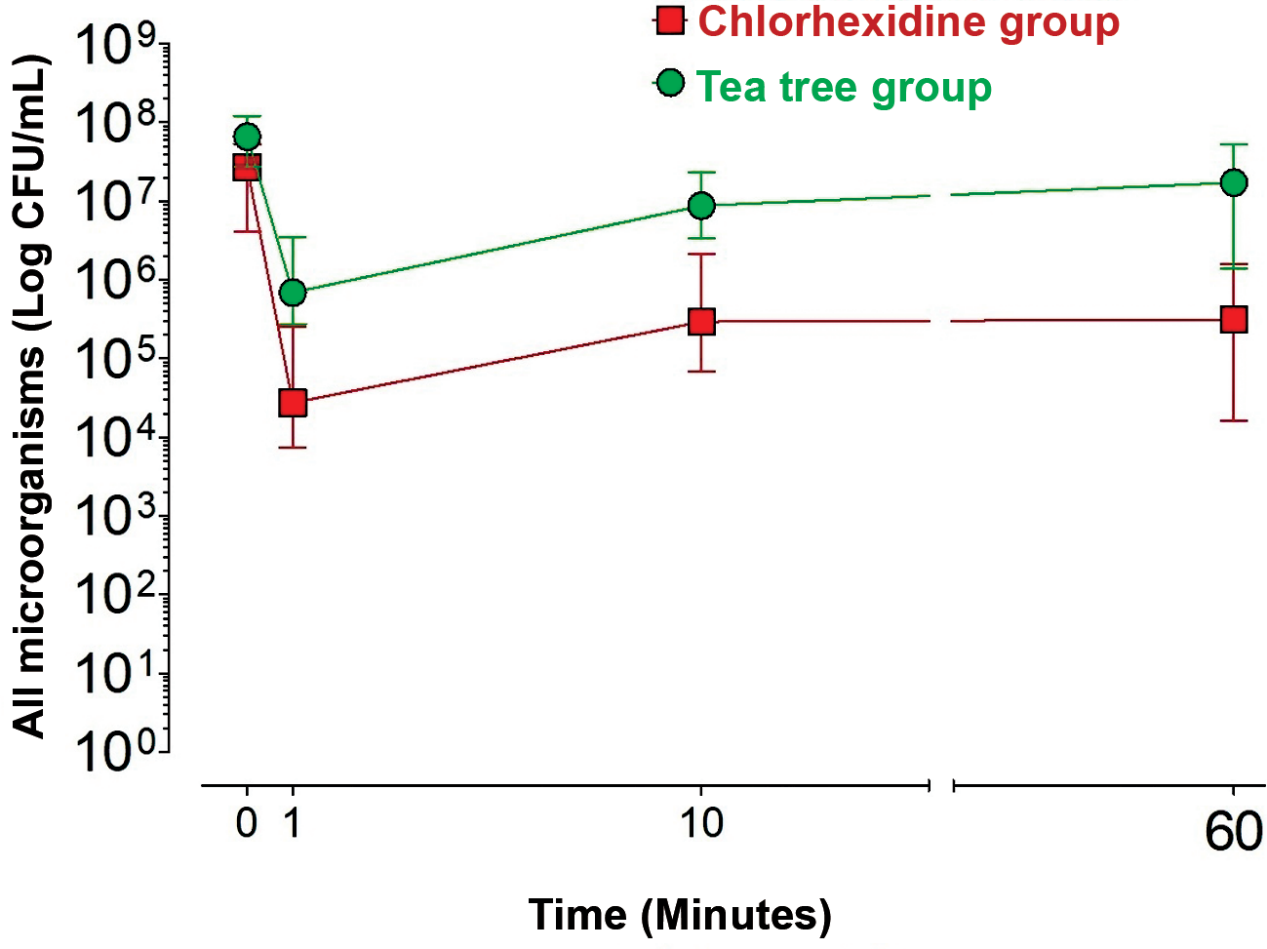
The median values of colony forming unit (CFU/mL) of all microorganisms (Brain Heart Infusion medium) according to treatment and time.

In the chlorhexidine group, there was a significant decrease in the number of colonies of all microorganisms at T1, T10, or T60 compared with T0 ( *p* < 0.05). Furthermore, there were no significant differences between T1, T10, and T60 ( *p* > 0.05; Fig. 2 ).

### Tea Tree Group (BHI Medium)

In the tea tree group, there was a significant reduction in the number of colonies of all microorganisms at T1 or T10 when compared with T0 ( *p* < 0.05). There was no significant difference between T1 and T10 ( *p* > 0.05). There was a significant increase in the number of colonies of all microorganisms at T60 when compared with T1 or T10 ( *p* < 0.05), and there was no significant difference between T0 and T60 ( *p* > 0.05; Fig. 2 ).

### Chlorhexidine Group versus Tea Tree Group (BHI Medium)

At T0, the chlorhexidine group showed a significantly lower value of CFU/mL compared with the tea tree group ( *p* < 0.05). At T1, T10, and T60, the chlorhexidine group showed significantly lower CFU/mL values compared with the tea tree group ( *p* <0.05; Fig. 2 ).

## Discussion

This study evaluated *Streptococcus mutans* present in the saliva of patients who received dental implants for two reasons. First, *Streptococcus mutans* is considered one of the main human cariogenic agents. 18 19 According Lemos et al, it is accepted that the cariogenic potential of *Streptococcus mutans* resides in three attributes: 19 “(I) the ability to synthesize large quantities of extracellular polymers of glucan from sucrose that aid in the permanent colonization of hard surfaces and in the development of the extracellular polymeric matrix *in situ* , (II) the ability to transport and metabolize a wide range of carbohydrates into organic acids (acidogenicity), and (III) the ability to thrive under environmental stress conditions, particularly low pH (aciduricity).” 19 And second, the oral biofilm formed by *Streptococcus mutans* can accumulate on dental implants causing failure of their osseointegration. 16 It is noteworthy that, as this microorganism creates a rich extracellular polysaccharide matrix and a low pH local environment, this can generate an increase in the number of other acidogenic and aciduric microorganisms, 19 which can also cause failure of implant osseointegration.

In the human mouth, *Streptococcus mutans* are usually detected and enumerated from saliva samples, dental plaque samples, or both. 18 Despite this, *Streptococcus mutans* colony counts are usually done from saliva samples, which are easier to obtain than dental plaque. 18 Levels of this microorganism in human saline reflect the number of colonized intraoral sites. 8 18 Thus, the use of saliva samples is justified.

The chlorhexidine group showed significantly lower CFU/mL values for *Streptococcus mutans* at T1, T10, and T60 compared with the tea tree group ( *p* < 0.05). Furthermore, chlorhexidine generated a significant reduction in the number of colonies of this microorganism at T1, T10, or T60 compared with T0, unlike tea tree oil, which significantly reduced the number of colonies of this microorganism only at T1 (T0 compared with T1). Thus, according to the present study, 0.12% chlorhexidine is more indicated as a preoperative mouthwash than 0.2% tea tree oil for two reasons: (I) its effect against *Streptococcus mutans* was superior to tea tree oil; and (II) chlorhexidine maintained its effect of reducing the number of *Streptococcus mutans* colonies throughout the surgical period (T10 or T60 < T0, *p* < 0.05), which can prevent infections generated by this pathogen. Tea tree oil, on the other hand, lost its effect of reducing the number of colonies of this pathogen throughout the surgery (T1 < T10, T60 or T0, *p* < 0.05; T10 or T60 = T0, *p* > 0.05). It is noteworthy that a significant reduction in the number of *Streptococcus mutans* has been considered equivalent to a decrease in the number of oral diseases (e.g., caries and peri-implant disease). 8

For the evaluation of all microorganisms detected by the BHI culture medium, the chlorhexidine group showed significantly lower values of CFU/mL at T1, T10, and T60 compared with the tea tree group. This again shows that 0.12% chlorhexidine has a greater antimicrobial effect than 0.2% tea tree oil. In addition, chlorhexidine maintained its effect in reducing the number of colonies of microorganisms throughout the surgical period (T10 or T60 < T0, *p* < 0.05), unlike tea tree oil, which during the surgical period maintained its effect in reducing the number of colonies of microorganisms only at T10 (T10 < T0, *p* < 0.05; T60 = T0, *p* > 0.05). Therefore, based on these results, chlorhexidine is even more indicated as a preoperative mouthwash. It is worth remembering that chlorhexidine, in this study, was used as a preoperative mouthwash and, therefore, there was no concern about its disadvantageous effects that may occur when this substance is used for long periods of time (e.g., dental dyschromia and taste alteration). 2 7 10 11 13

Limitations of this study include the use of only two types of mouthwashes, as well as the maximum evaluation time, which was only 61 minutes. Further studies of this nature are recommended using tea tree oil in other clinical situations.

## Conclusion

Chlorhexidine is more indicated as a preoperative mouthwash than tea tree oil, due to its significantly more effective antimicrobial action.
